# Diagnostic methods to determine microbiology of postpartum endometritis in South Asia: laboratory methods protocol used in the Postpartum Sepsis Study: a prospective cohort study

**DOI:** 10.1186/s12978-016-0121-4

**Published:** 2016-02-25

**Authors:** Sadia Shakoor, Megan E. Reller, Amnesty LeFevre, Aneeta Hotwani, Shahida M. Qureshi, Farheen Yousuf, Mohammad Shahidul Islam, Nicholas Connor, Iftekhar Rafiqullah, Fatima Mir, Shabina Arif, Sajid Soofi, Linda A. Bartlett, Samir Saha, the ANISA-Postpartum Sepsis Study Group

**Affiliations:** Department of Paediatrics and Child Health, Division of Women & Child Health, The Aga Khan University, Stadium Road, P.O. Box 3500, Karachi, Pakistan; Department of Pathology and Laboratory Medicine, The Aga Khan University, Stadium Road, P.O. Box 3500, Karachi, Pakistan; Department of Obstetrics and Gynecology, Division of Women & Child Health, The Aga Khan University, Stadium Road, P.O. Box 3500, Karachi, Pakistan; Division of Medical Microbiology, Department of Pathology, The Johns Hopkins University School of Medicine, 615 N. Wolfe Street, Baltimore, MD USA; Department of International Health, Johns Hopkins Bloomberg School of Public Health, 615 N. Wolfe Street, Baltimore, MD USA; The Child Health Research Foundation, Department of Microbiology, Dhaka Shishu Hospital, Sher-E-Banglanagar, Dhaka, Bangladesh

**Keywords:** Puerperal sepsis, Postpartum, Etiology, Microbiology, Pakistan, Bangladesh

## Abstract

**Background:**

The South Asian region has the second highest risk of maternal death in the world. To prevent maternal deaths due to sepsis and to decrease the maternal mortality ratio as per the World Health Organization Millenium Development Goals, a better understanding of the etiology of endometritis and related sepsis is required. We describe microbiological laboratory methods used in the maternal Postpartum Sepsis Study, which was conducted in Bangladesh and Pakistan, two populous countries in South Asia.

**Methods/Design:**

Postpartum maternal fever in the community was evaluated by a physician and blood and urine were collected for routine analysis and culture. If endometritis was suspected, an endometrial brush sample was collected in the hospital for aerobic and anaerobic culture and molecular detection of bacterial etiologic agents (previously identified and/or plausible).

**Discussion:**

The results emanating from this study will provide microbiologic evidence of the etiology and susceptibility pattern of agents recovered from patients with postpartum fever in South Asia, data critical for the development of evidence-based algorithms for management of postpartum fever in the region.

## Background

Puerperal pyrexia and post-partum puerperal (PP) sepsis, an infection acquired during the first 42 days postpartum, remain leading causes of maternal morbidity and mortality in South Asia [1]. It is estimated that >500, 000 women die of complications of pregnancy and childbirth. The Millennium Declaration had pledged to decrease all-cause maternal mortality as part of its Millennium Development Goal 5 (MDG-5); however, the 2014 report states that the target to reduce the maternal mortality rate by three quarters has not been met [2]. The puerperal sepsis (PP Sepsis) study, a supplement to the Gates Foundation-funded Aetiology of Neonatal Infections in South Asia (ANISA) study, was undertaken to improve the very high rate of maternal mortality in South Asia by delineating the infectious causes of fever in this population and validating related clinical algorithms for management of PP sepsis in the community.

This paper describes the samples collected and diagnostic tests used to determine and characterize the bacterial etiologies of severe postpartum infections in study centers in Pakistan (Matiari and Karachi) and Bangladesh (Sylhet). Quality assurance procedures at each site are also described.

## Methods/Design

### The primary PP Sepsis Study

The PP sepsis study is a prospective cohort study that expands on the Aetiology of Neonatal Infection in South Asia (ANISA) study in three sites of Sylhet in Bangladesh and Karachi and Matiari in Pakistan. Methods for field evaluation are described by Bartlett et al. [3]. Briefly, through ten scheduled home visits made by Community Health Workers in these sites, postpartum mothers were evaluated according to a modified diagnostic algorithm to identify puerperal sepsis as a cause of postpartum fever and then assessed by a study physician. After informed written consent, clinical diagnostic specimens, including urine, blood, and endometrial, were collected from these confirmed puerperal sepsis cases. All women with puerperal sepsis were prescribed recommended first-line antibiotics (amoxicillin-clavulanate or oral clindamycin with intramuscular gentamicin) based on a recent metaanalysis [4], unless otherwise indicated by the laboratory results.

The study protocol was approved after full ethical review by the Johns Hopkins University, Baltimore, USA; International Centre for Diarrheal Disease Research, Bangladesh; the Bangladesh Institute for Child Health for the Child Health Research Foundation in Bangladesh, and the Aga Khan University, Karachi, Pakistan.

### Laboratory network for the PP Sepsis study

Microbiological and other laboratory tests were carried out at research laboratories in Karachi, Sylhet, and Dhaka. The Infectious Disease Research Laboratory (IDRL) at the Aga Khan University received and processed specimens from the Karachi and Matiari field sites, whereas, the Child Health Research Foundation (CHRF) Microbiology Laboratory at the Dhaka Shishu Hospital received specimens from the Sylhet site.

### Choice of diagnostic specimens for investigation of puerperal sepsis

The WHO advises culture of midstream urine specimens and blood for evaluation of the cause of post-partum maternal fever [5]. Transvaginal aspirates of endometrium or endometrial tissue specimens are also advised by the American Society for Microbiology for confirmation of endometritis and delineation of the organisms responsible for it [6].

In keeping with these recommendations, we collected the following specimens to evaluate the cause of postpartum fever, following informed consent.

#### Blood

Blood was collected for complete blood count, culture, and to determine the presence or absence of malarial parasites. The purpose of each of these tests was unique and has been described in Table 1.

**Table 1:** Samples for evaluation of Postpartum sepsis in the PP Sepsis Study

Type of Specimen	Methods	Collection protocol/ transport protocol and times	Target pathogens	Purpose
Blood	Blood count by flow cytometry	Collection in EDTA tube, transport at 2–8 °C, within 4–6 h to laboratory	None	Leukocytosis or leukopenia and thrombocytopenia as parameters of sepsis
	Peripheral blood microscopy for malarial parasites	As above	*Plasmodium* spp (*vivax* and *falciparum*)	Exclusion of malaria as a cause of postpartum fever
	Culture (aerobic and anaerobic)	Collection in Aerobic and Anaerobic BACTEC blood culture bottles, room temperature, within 4–6 h to laboratory	Bacteria/ fungi	Culture of blood for target pathogens of endometritis
Urine	Pyuria by leukocyte esterase Bacteria by nitrite	Collection in boricon-containing wide-mouthed bottles, transport at room temperature, within 4–6 h to laboratory	None	Evaluation of urine as a surrogate specimen for diagnosis of endometritis; correlation with endometrial culture results
	Quantitative culture	As above	Bacteria and yeasts	
Endometrium	Semiquantitative culture NAAT	Collection in thioglycollate medium, room temperature, within 2–4 h Collection in Universal Transport Medium, transport at 2-8 ° C within 2–4 h to laboratory	Bacteria, yeasts *Chlamydia*/ *Mycoplasma*/ *Ureaplasma*	Reference sample for bacterial pathogens of endometritis
High vaginal swab	Semiquantitative culture	Collection in Ames transport medium swabs, within 4–6 h, at room temperature	Bacteria, yeasts	Comparison of HVS colonizing flora to evaluate and exclude contamination of vaginal flora from endometrial specimens

To evaluate for leukocytosis and leukopenia as possible markers of sepsis [7], blood counts were determined by flow cytometry at central laboratories affiliated with field sites. Blood samples of at least 1 ml were collected in ethylene diamine tetra-acetate (EDTA) tubes and transported to laboratories at 2–8 °C within 6 h of collection.

Thin and thick blood smears were made and examined at central laboratories to identify malarial parasites and to quantify and speciate Plasmodia, if present.

Aerobic and anaerobic blood cultures were performed in the BACTEC 9240® instrument with BACTEC Aerobic Plus® and Anaerobic F Lytic bottles. Five to ten ml of blood was collected in each bottle and transported to respective central laboratories for incubation and processing. Those flagged positive by the instrument were removed and gram stain and agar subculture was performed.

#### Endometrial specimen collection

**Fig. 1:**
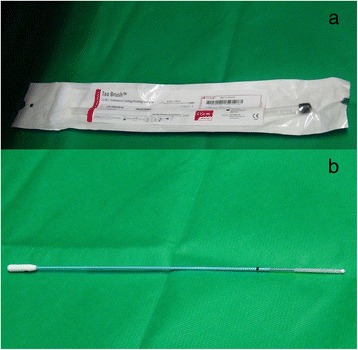
The Cook Medical Tao brush for endometrial specimen collection (a) with and (b) without packaging

Endometrial tissue was collected using Tao sampling brushes by Cook Medical [8]. Sterile brushes (Fig. 1) were opened tail-end first immediately after insertion of the Cusco speculum and visualization of the cervix. Manufacturer’s instructions were followed to collect the endometrial tissue on the brush. Tao brushes are designed to collect endometrial tissue for cytology and histopathology. In using the same brush for culture, we modified the instructions to avoid vaginal contamination. The rounded tip at the sampling end was snapped off with sterile scissors. The brush was then cut into 2 halves and one half was used for endometrial culture and the other reserved for molecular detection of additional potential pathogens (bacteria not readily cultivable). A supplementary video of collection procedures as a mock demonstration is available as Supplementary File 1.

#### Endometrial culture

One half of the brush was aseptically cut and placed into a thioglycolate broth medium with cooked meat to facilitate recovery of strict anaerobes. This tube was transported to the central laboratory at 25 °C within 6 h of collection. Upon receipt in the laboratory, the broth was vortexed at high speed to dislodge tissue from the brush and used to inoculate chocolate, MacConkey, and 5 % sheep blood agar (SBA) media. To recover strict anaerobes, pre-reduced Brucella agar with kanamycin and vancomycin supplements was inoculated with the thioglycolate broth [9]. Agar incubation temperatures, sources, and culture methods and result parameters are described in detail in Table 2. Results were recorded semi-quantitatively as light (few colonies), medium, or heavy growth. Identification of gram-negative organisms was carried out with commercial API systems (BioMerieux) strips, (API 20E for enteric gram negative bacteria and API NE for non-fermenting gram negative bacteria). Identification of gram positive bacteria was based on the Clinical Laboratory Standards Institute (CLSI)-recommended tests [10], such as tube coagulase for *S.aureus* and bacitracin disk and Lancefield grouping for *S.pyogenes* and *S.agalactiae*. Commercial systems such as API Strep and API-NH (BioMerieux) were used for fastidious organisms, such as alpha haemolytic, nutritionally variant *Streptococci,* and *Haemophilus* spp.

**Table 2:** Agar media, target pathogens and incubation protocols for endometrial cultures in the PP Sepsis Study

Culture media	Target pathogen(s)	Incubation atmosphere, temperature, and time	Manufacturer
Chocolate agar	Gram positive and negative aerobes and facultative anaerobes; fastidious bacteria (eg *Haemophilus* spp)	37 °C in 5–10 % CO_2_ for 48 h	Oxoid^a^/Isovitalex BD^b^
5 % sheep blood agar	Gram positive and negative aerobes and facultative anaerobes, yeasts	37 °C in 5–10 % CO2 for 48 h	Oxoid
Maconkey agar	Fermenting and non-fermenting gram negative bacilli	37 °C in ambient air for 48 h	Oxoid
Bilayer colistin/ nalidixic acid agar with 5 % sheep blood	Selective for gram positive pathogens (eg *S.aureus*, streptococci)	37 °C in 5–10 % CO_2_ for 48 h	Oxoid
Banked human blood-tween agar	*G.vaginalis*	37 °C in 5–10 % CO_2_ for 48 h	Oxoid/Tween 80 Sigma^c^
Gonococcal agar	*Neisseria gonorrheae*	37 C in 5–10 % CO_2_	Oxoid (VCAT^d^ supplement from oxoid)
Anaerobic agars: Schaedler Agar with Vitamin K1 and 5 % Sheep Blood Brucella Blood Agar with Hemin and Vitamin K1 Phenylethyl alcohol-SBA (PEA-SBA)	Anaerobes	Anaerobic atmosphere, 35 ± 2 °C for 72 h	Oxoid/Vit K Sigma Fluka^e^/Vit K and Hemin Sigma BD/ supplements from Sigma

^a^Oxoid, Thermo Fisher Scientific Inc., UK

^b^BD = Becton Dickinson, NJ, USA

^c^Sigma-Aldrich, St Louis, MO, USA

^d^VCAT = Vancomycin-chloramphenicol-amphotericin-trimethoprim

^e^Fluka Analytical, Hanover, German

#### Molecular detection of difficult-to-culture pathogens

The other half of the endometrial brush was introduced into a Universal Transport Medium (UTM) tube (Copan) and transported to the laboratory at 2–8 °C.

The presence of *Chlamydia trachomatis*, *Mycoplasma* spp, and *Ureaplasma* spp was detected by molecular methods. For specimens collected in Pakistan, the Sacace Biotechnologies (Como, Italy) kit [11] was used to identify the presence of *C.trachomatis*, *M.genitalium*, *M.hominis*, and *U.urealyticum* at Aga Khan University’s Infectious Disease Research Laboratory. Endometrial specimens obtained in Bangladesh were shipped to University of Alabama (UAB) for culture of mycoplasma and ureaplasma and PCR detection for *Mycoplasma* and *Ureaplasma* using previously published methods [12].

In Pakistan, high vaginal swabs were collected after endometrial brush specimens in an attempt to exclude vaginal contaminating flora and to evaluate for presence of vaginitis on the basis of presence or absence of clue cells. Samples were collected from the high vaginal wall in Ames transport medium (Medical Wire Transwab Ames, UK) and sent to the laboratory. Samples were plated on chocolate agar, Gardnerella agar (with 5 % human blood), Maconkey agar, and Sabouraud’s agar. Results were recorded semi-quantitatively as light, medium, or heavy growth.

#### Urine

Clean catch urine was collected to determine the etiology of urinary tract infection (UTI) in patients with likely UTI (significant growth of uropathogen with positive nitrite and leukocyte esterase on dipstick test) [13].

Dipstick tests, indicating the presence or absence of both leukocyte esterase and nitrite [12] were performed to identify supportive evidence of UTI. Quantitative cultures of urine were performed for growth of uropathogens on SBA and MaConkey’s agar media, and ≥ 10^3^ colony-forming unit (CFU) of bacteria per mL of urine were considered significant. Identification of uropathogens was carried out using either the minimal sulfide-indole-motility-citrate-urease-triple sugar iron (SIMCUT) method or commercial API (BioMerieux) strips.

#### Susceptibility testing of pathogens

Susceptibility testing by the disk diffusion method was carried out only for aerobic or facultatively anaerobic pathogens in accordance with CLSI guidelines. Where disk diffusion was not the preferred method, broth dilution minimum inhibitory concentrations (MICs) were carried out to confirm susceptibility (eg for methicillin resistant *S.aureus*, vancomycin MICs were performed by the broth dilution method) [14].

#### Ancillary diagnostic tests for fever

At the Karachi site, dengue IgM and Rapid ICT malaria (PanBio) tests were additionally performed to detect dengue fever, and to improve detection of submicroscopic malaria infections. The city suffered major dengue outbreaks since 1994 [15], and continues to experience annual outbreaks [16].

### Quality assurance

The Pakistan site research laboratory regularly undergoes biannual proficiency testing (PT) for bacteriologic culture and identification and molecular detection of respiratory pathogens by the College of American Pathologists (CAP). The laboratory achieved 98 % satisfactory results for culture and identification and 95 % satisfactory scores for molecular detection and identification of pathogens in 2013.

At the beginning of the study, the lead technologist at the Bangladesh site visited Johns Hopkins Medical Institutions clinical microbiology laboratory to review procedures and for additional specialized training. Research laboratories in Sylhet and Dhaka carried out isolation and identification and sent 10 % of bacterial pathogens isolated from blood to Infectious Disease Research Laboratory in Karachi to confirm identification.

### Microbiologic test review and interpretation, and consolidation

Procedures for and results of laboratory tests have been reviewed by experienced and certified clinical microbiologists at both sites (SS and MER). Results were also reviewed mid-study in November 2013 and at end-of-study in November 2014 at the PP sepsis Technical Advisory Group Meeting.

Although there were minor variations in test menus (ancillary tests carried out for Karachi sites) and differences in quality assurance procedures (IDRL subscribes to CAP PT, while Sylhet laboratory results were verified by IDRL at the Aga Khan University and by UAB), tests at either site laboratory were carried out using the same Standard Operation Procedures (SOPs) developed by SS and MER and reviewed by other members of the team.

## Discussion

In implementing protocols and carrying out tests that had to be reported to physicians in time, the role of the laboratory in reporting timely and reliable results cannot be overemphasized. Laboratory managers played key roles in contacting study physicians and principal investigators with timely results. Among the challenges faced were difficult maintenance of transport temperatures of samples (different halves of the same endometrial brush had to be transported both at room temperature and refrigerated temperature), transport of laboratory samples over long distances, and timely transport. Upon interim review of laboratory data, it was noted that earlier processing of cultures resulted in fewer contaminants and desirable colony counts. Thus, management of employee shifts to adjust culture processing times from 4 to 6 h lags to immediate processing was carried out.

In conclusion, laboratory protocols for the etiology of postpartum fever study, as described, performed smoothly and efficiently despite challenges of implementation. Routine collection of endometrial samples in the community must be complemented by efficient laboratory services for optimal recovery of pathogens.

## Abbreviations

PP: Postpartum or puerperal
MDG: Millennium Development Goals
ANISA: Aetoiology of Neonatal Infection in South Asia Study
CHRF: Child Research Foundation
WHO: World Health Organization
EDTA: Ethylene-diamine tetra-acetate
SBA: Sheep blood agar
API: Analytical profile index
CLSI: Clinical Laboratory Standards Institute
UTM: Universal transport medium
PCR: Polymerase chain reaction
UTI: Urinary tract infection
CFU: Colony-forming unit
SIMCUT: Sulfide-indole-motility-citrate-urease-triple sugar iron method
MIC: Minimal inhibitory concentration
PT: Proficiency testing
ICT: Immunochromatographic Test
CAP: College of American Pathologists
IDRL: Infectious Disease Research Laboratory
SOP: Standard operating procedures
UAB: University of Alabama
